# Correction to: A genome sequence for the threatened whitebark pine

**DOI:** 10.1093/g3journal/jkae085

**Published:** 2024-04-29

This is a correction to: David B Neale, Aleksey V Zimin, Amy Meltzer, Akriti Bhattarai, Maurice Amee, Laura Figueroa Corona, Brian J Allen, Daniela Puiu, Jessica Wright, Amanda R De La Torre, Patrick E McGuire, Winston Timp, Steven L Salzberg, Jill L Wegrzyn, A genome sequence for the threatened whitebark pine, G3 Genes|Genomes|Genetics, 2024;, jkae061, https://doi.org/10.1093/g3journal/jkae061

In the originally published version of this article, one row of Table 4, with statistics for the genome of Engelmann spruce, was inadvertently omitted, but the spruce genome statistics were used in the row for Siberian larch, instead of the correct statistics for that genome. The following corrected table 1) provides the correct statistics for the Siberian larch genome and cites the original paper reporting them, 2) adds the row for the Engelmann spruce genome and cites the NCBI BioProject source for those otherwise unpublished statistics, and 3) adjusts the footnote providing the citations for these genomes.

**Table 4.** Quantitative statistics of recently published large conifer genomes

Genome assembly [Source]^a^	Total sequence (Gbp)	N50 contig size (bp)	N50 scaffold size (bp)	Number of contigs	Number of scaffolds	Sequencing strategy
Douglas-fir v1.0 [1]	14.4	67,133	381,586	1,726,175	1,236,665	Illumina
Sugar pine v1.5 [2]	25.5	7,947	292,700	14,950,590	4,253,097	Illumina+10x
Loblolly pine v2.0 [3]	20.5	28,106	107,038	2,724,159	1,760,464	Illumina+ PacBio RSII
Giant sequoia v2.0 [4]	8.1	318,087	690,549,816	52,835	8,216	Illumina+ONT +HiC
Coast redwood v1.0 [5]	26.5	97,162	45,116,641	548,918	393,407	Illumina+ONT +HiC
Siberian larch [6]	7.9	1,064	6,479	12,480,170	11,197,034	Illumina
White spruce [7]	21.6	9,736	161,389	3,849,851	2,445,336	Illumina+10x
Japanese larch [8]	13.0	447,849	447,849	65,219	65,219	PacBio
Chinese pine [9]	25.4	2,601,037	2,107,674,557	22,739	7,371	Illumina+PacBio CLR+HiC
Engelmann spruce [10]	20.7	17,914	403,791	2,394,260	946,236	Illumina+ONT +10x
Whitebark pine v1.0 [11]	27.6	537,007	2,005,774,401	92,740	34,176	Illumina+ONT +OmniC

^a^Sources: 1=Neale *et al.* (2017); 2=Crepeau *et al.* (2017); 3=Zimin *et al.* (2017b); 4=Scott *et al.* (2020); 5=Neale *et al.* (2022); 6=Kuzmin *et al.* (2019); 7=Gao *et al.* (2022); 8=Sun *et al.* (2022); 9=Niu *et al.* (2022); 10=NCBI BioProject PRJNA504036; 11=this paper.

Table 4 has been corrected in the original article and the following reference has been added to the “Literature cited” section:

Kuzmin DA, Feranchuk SI, Sharov VV, Cybin AN, Makolov SV, Putintseva YA, Oreshkova NV, Krutovsky KV. 2019. Stepwise large genome assembly approach: a case of Siberian larch (*Larix sibirica* Ledeb). *BMC Bioinformatics 20*:37. https://doi.org/10.1186/s12859-018-2570-y

