# Supplementary material for: A genome sequence for the threatened whitebark pine
Source: G3 (Bethesda). 2024 Mar 25;14(5):jkae061. doi: 10.1093/g3journal/jkae061 (PMC11075562; doi:10.1093/g3journal/jkae061)
Supplement: jkae061_Supplementary_Data [file jkae061_supplementary_data.docx]

**Supplementary tables**

**Table S1. Stocks for library preparation protocol for ONT long-read sequencing**

| **Reagent** | **Volume** |
| --- | --- |
| **HB (Homogenization Buffer) Stock 10X (100 mL)** | |
| Trizma base | 1.21 g |
| Potassium chloride (KCl) | 5.96 g |
| 0.5 M EDTA | 20 mL |
| Spermidine | 0.255 g |
| Spermine | 0.348 g |
| Bring to 100 mL with double-distilled H_2_O (ddH_2_O), adjust pH to 9.0 to 9.4 with 10 M NaOH drops. This stock can be stored at 4°C in a glass bottle for 3 months. | |
| **HB 1X Solution (500 mL)** | |
| 10X HB | 50 mL |
| Sucrose | 85.6 g |
| Bring to 350 mL with ddH_2_O, stir until dissolved. Bring final volume to 500 mL with ddH_2_O. This stock can be stored at 4°C in a glass bottle for 3 months. | |
| **Triton X-100 (20% vol/vol, 100 mL)** | |
| Triton X-100 | 20 mL |
| 10X HB | 10 mL |
| Sucrose | 17.15 g |
| Bring to 60 mL with ddH_2_O, stir until dissolved. Bring final volume to 100 mL with ddH_2_O. This stock can be stored at 4°C in a glass bottle for up to 1 year. | |
| **NIB (Nuclear Isolation Buffer)** | |
| 1X HB | 48.75 mL |
| Triton X-100 mix | 1.25 mL |
| Polyvinylpyrrdidone (PVP) | 0.5 g |
| This buffer should be prepared the day before isolation. The PVP takes approximately 5 to 7 hours to dissolve. | |

**Table S2. Summary statistics for transcriptomic evidence used for gene prediction (reads only)**

| **SRA accession** | **BioProject** | **Tissue type** | **Raw reads (M)** | **Trimmed reads (M)** | **Mapping rate (%, v0.9)** | **Mapping rate (%, v1.0)** |
| --- | --- | --- | --- | --- | --- | --- |
| SRR13823648 | PRJNA703422 | megagametophyte | 68.9 | 66.9 | 95.2 | 93.8 |
| SRR5368574 | PRJNA352055 | needle | 38.0 | 37.2 | 96 | 94.6 |
| SRR5368575 | PRJNA352055 | needle | 41.2 | 40.3 | 95.9 | 94.6 |
| SRR5368576 | PRJNA352055 | needle | 37.95 | 37.1 | 95.3 | 93.8 |
| SRR5368577 | PRJNA352055 | needle | 35.2 | 34.2 | 95.3 | 93.7 |
| SRR5368578 | PRJNA352055 | needle | 34.3 | 33.5 | 95.5 | 93.9 |
| SRR5368579 | PRJNA352055 | needle | 32.3 | 31.4 | 96 | 94.7 |
| SRR5368580 | PRJNA352055 | needle | 25.20 | 24.6 | 96 | 94.5 |
| SRR5368581 | PRJNA352055 | needle | 30.6 | 29.95 | 96 | 94.5 |
| SRR5368582 | PRJNA352055 | needle | 24.4 | 23.9 | 94.8 | 93.3 |
| SRR4786281 | PRJNA352055 | needle | 36.1 | 35.1 | 95 | 93.3 |
| SRR4786284 | PRJNA352055 | needle | 46.2 | 44.5 | 94.8 | 93 |

**Table S3. Summary statistics for the de novo transcriptome used as full-length evidence**

|  | **Lib 1** | **Lib 2** | **Lib 3** | **Lib 4** | **Lib 5** | **Lib 6** | **ORP reference** |
| --- | --- | --- | --- | --- | --- | --- | --- |
| Trinity | | | | | | | |
| Transcripts | 39112 | 40849 | 39405 | 34763 | 43704 | 40301 | 139837 |
| N50 | 2605 | 1297 | 2759 | 505 | 1682 | 2908 | 2882 |
| Transcripts2 | 45594 | 47635 | 44799 | 39359 | 52025 | 44891 | 86369 |
| N50 | 2250 | 321 | 498 | 939 | 1764 | 774 | 726 |
| Complete BUSCOs | 60.40% | 73.60% | 61.70% | 59.70% | 79.10% | 60.90% | 88.50% |
| Single-copy BUSCOs | 39.50% | 49.40% | 41.50% | 42.20% | 50.70% | 38.50% | 46.60% |
| Duplicated BUSCOs | 20.90% | 24.20% | 20.20% | 17.50% | 28.40% | 22.40% | 41.90% |
| Fragmented BUSCOs | 12.40% | 8.70% | 12.60% | 12.70% | 6.40% | 12.30% | 2.90% |
| Missing BUSCOs | 27.20% | 17.70% | 25.70% | 27.60% | 14.50% | 26.80% | 8.60% |
| EnTAP | | | | | | | |
| Total sequences | 23926 | 24857 | 23604 | 21969 | 26085 | 23021 | 66233 |
| N50 | 1218 | 1419 | 408 | 381 | 375 | 1203 | 2217 |
| Similarity search | 14404 | 15159 | 14483 | 13545 | 16152 | 14044 | 43180 |
| Total unique sequences with family assignment | 18902 | 19387 | 18801 | 17840 | 20444 | 18800 | 56732 |
| Total unique sequences with at least one GO term | 16417 | 16579 | 16290 | 15436 | 17328 | 16427 | 48221 |
| Total unique sequences with at least one pathway (KEGG) assignment | 5243 | 5099 | 5217 | 4928 | 5169 | 5358 | 14165 |
| Total unique sequences annotated (gene family and/or similarity search) | 18917 | 19411 | 18818 | 17854 | 20465 | 18817 | 56796 |
| Mapping stats Minimap | | | | | | | |
| Total reads |  | | | | | | 694514 |
| Secondary |  | | | | | | 187758 |
| Supplementary |  | | | | | | 43125 |
| Duplicates |  | | | | | | 0 |
| Mapped |  | | | | | | 498039 (71.71%) |

**Table S4.** **Repeat content**

|  |  | **Total elements** | **Element length (bp)** | **Coverage (%)** |
| --- | --- | --- | --- | --- |
| SINEs |  | 0 | 0 | 0 |
| LINEs |  | 702,573 | 578,885,792 | 2.09 |
|  | LINE1 | 370,868 | 404,306,289 | 1.46 |
| LTR |  | 7,347,127 | 11,559,544,152 | 41.77 |
|  | ERV_classII | 42,505 | 35,061,926 | 0.13 |
| DNA |  | 399,110 | 310,688,087 | 1.12 |
| Unclassified |  | 18,838,504 | 8,937,651,555 | 32.295 |
| Total interspersed |  |  | 21,386,769,586 | 77.28 |
| Small RNA |  | 45,706 | 40,193,439 | 0.145 |
| Simple repeats |  | 44,012 | 46,361,512 | 0.17 |
| Total |  |  | 21,473,324,537 | 77.59 |

**Table S5. Genome annotation approaches**

| **Run** | **Total transcripts** | **Total genes** | **Gene N50 (bp)** | **BUSCO (run in protein mode)^a^** | **EnTAP (%)** | **Mono:multi** | **Max intron (Mb)** | **average exons per gene** |
| --- | --- | --- | --- | --- | --- | --- | --- | --- |
| StringTie2 (SR) | 63,123 | 51,844 | 2,281 | C:70.9% [S:43.3%, D:27.6%], F:15.2%, M:13.9% | - | 0.34 | 1.39 | 4.7 |
| StringTie2 (Hybrid) | 62,936 | 47,132 | 1,807 | C:71.4% [S:37.4%, D:34.0%], F:16.5%, M:12.1% | - | 0.33 | 1.02 | 4.2 |
| BRAKER (SR) | 636,628 | 631,708 | 963 | C:45.0% [S:35.6%, D:9.4%], F:30.8%, M:24.2% | - | 1.03 | 0.14 | 2 |
| BRAKER (SR) eggNOG Filtered | 219,474 | 216,079 | 1,164 | C:45.0% [S:35.7%, D:9.3%], F:30.9%, M:24.1% | - | 0.70 | 0.14 | 2.7 |
| StringTie2 (SR) eggNOG + Transdecoder filtered | 48,567 | 27,336 | 1,578 | C:70.5% [S:43.2%, D:27.3%], F:15.1%, M:14.4% | 0.85 | 0.22 | 1.39 | 5.9 |
| StringTie2 (Hybrid) eggNOG + Transdecoder filtered | 45,380 | 31,953 | 1,515 | C:70.3% [S:45.6%, D:24.7%], F:16.0%, M:13.7% | 0.81 | 0.23 | 1.02 | 5.3 |
| v0.9 final annotation | 47,911 | 27,010 | 1,578 | C:70.6% [S:43.2%, D:27.4%], F:15.1%, M:14.3% | 0.87 | 0.22 | 1.39 | 6 |
| v1.0 final annotation | 58,831 | 27,555 | 1,590 | C:73.9% [S:21.7%, D:52.2%], F:5.5%, M:20.6% | 0.71 | 0.33 | 2.45 | 7.5 |

^a^C=Complete; S=Single copy; D=Duplicated; F=Fragmented; M=Missing

**Table S6. Summary statistics for v0.9 NLR identification**

|  | **Only NB-ARC** | **Missing N-terminal** | **Missing LRR** | **Complete** | **Total** |
| --- | --- | --- | --- | --- | --- |
| De novo assembled transcriptome | 27 | 24 | 14 | 24 | 89 |
| Direct genome (NLR-Annotator) | 616 | 771 | 257 | 595 | 2239 |
| Genome annotation | 56 | 57 | 37 | 88 | 238 |
| BRAKER gene model | 0 | 0 | 0 | 27 | 27 |
